# Mr. Leese, on Vaccination

**Published:** 1804-08-01

**Authors:** L. Leese

**Affiliations:** Copthall Court


### *To the Editors of the Medical and Phyfical Journal.*

GENTLEMEN,

I Had hoped that the war of words had been no longer an incumbent duty on the believers and propagators of that great blessing to mankind, promulgated by Jenner; and, and, that the lancet only, and not the pen, was the sole instrument needful in the dissemination of their principles. I thought there could be no longer enemies to its general establishment, but such as were inaccessible, from refusing (through prejudice) the aid of their senses in informing their judgment. To have persevered in contention with such, would have been 'waging an inglorious war;' they might, without dereliction of duty, been left to have suffered by themselves or kindred, that merciless affliction for which the cow-pock is so mild and safe a substitute.—But the pamphlet of Mr. Goldson, and the opinion of a very respectable physician of this city, who had read that pamphlet, and who, to my concern and surprise, declares his belief, that those who have had cow-pock are liable to small-pox, and that the cow-pock does occasion peculiar and foul eruptions; shews that I was too sanguine in my expectations, and that it is yet necessary, that those who have had experience and conviction of its importance and utility should not be silent.

It is now four years since my doubts of the mildness and security of cow-pock have been subdued, by previously learning and observing all I could on the subject. Since that time, I have had considerable opportunities of confirming or invalidating my opinion, arising from being inoculator at one of the stations of the R. J. Society, in a very populous neighbourhood; from private practice, and from being a member of the medical council of the R. J. Society, the duty of which council it is to investigate all cases of vaccination attended with peculiar circumstances, whether arrising from coincidence of other diseases, suspicion of insecurity, imputation of small-pock succeeding cow-pock, or any consequences arising from vaccination; and having given all the attention I was able to all these points, I do most solemnly declare, I have not seen the small-pox take place after the cow-pock, nor have I ever seen the peculiar and foul eruptions, or any other arising from cow-pock.—But I have witnessed the most foul conduct among its adversaries; one out of many (because it is very recent) I will relate: A woman called with her child to consult me about vaccinating it, but her mind was not quite decided; because, she said, her husband was averse to it, and a neighbour of hers had a child now dangerously ill with the cow-pock. I assured her there was no danger from cow-pock, and I vaccinated the child. I asked her a few days after how the child was that was so ill of cow-pock; she answered, that since her own had been done, done, the *same* woman said her child had not been inoculated, and was ill from teething only. This appears to me to have been a contrivance between the woman who pretended her child had been vaccinated and the father of the other child, to prevent the vaccination of the one that was brought to me. This report would reach the ears of many whom the falsehood of it would not reach. The woman whose child I vaccinated, said the other had since thought of having hers done; but the medical gentleman told her the child would have breakings out spring and fall, so advised her not, but to have it inoculated with the *natural* sort.

In proof of the sincerity of my conviction, I have vaccinated my wife, two children and a female servant, in my own family.

With respect to Mr. Goldson's pamphlet, it is certainly interesting, and bespeaks a man who would write very creditably on any subject with which he was well acquainted, but he does not even pretend to have had much experience in cow-pock. His book is, therefore, either quite nugatory, or proves a great deal *too much.* Can any rational person believe so many cases of the small-pox supervening cow-pock can have happened, in a limited and private practice, to one practitioner, when none have manifested themselves to several who have had very extensive experience, both as public and private practitioners; for such is the testimony of Dr. Woodville, who, with his coadjutor, Mr. Wachsel, have vaccinated their tens of thousands; such is the testimony also of Dr. Jenner, Dr. Pearson, Mr, Ring, &c. &c. who have vaccinated their thousands.

The following is published under date of the 20th June, 1804, by the Society under the direction and inspection of Dr. Pearson.

" In the *last* fortnight, a number of subjects who had undergone vaccination in the year 1800, have been submitted to the test, or counter proof, (variolation) in circumstances the most favourable for exciting small-pox; also subjects who were vaccinated in Dr. Pearson's early practice, in 1799. The evidence from which demonstrates a uniform series of facts the reverse of Mr. Goldson's conclusions.

" What is the rational inference from all this, but that Mr. Goldson has been misguided by some fundamental error in his practice. But should it be found (though I believe it has not yet occurred) that one in many thousands has the small-pox after having had the perfect cow-pock, it it is no more than has happened after the small pox itself, according to the testimony of some of the most experienced practitioners; and the most ardent advocates for the cow-pock, do not pretend that it is a surer preventive than the small-pox itself. If it should continue to be urged, that the antidote only operates for a time, may I ask these gentlemen what time they mean? I flatter myself they will very soon be impelled by facts to name such a period, as not one in ten thousand of the youngest of our patients (though vaccinated) are likely to survive. There are many instances supported by most respectable testimony of persons resisting every known mode of communicating small-pox; ten, twenty, thirty, forty, and even fifty years after having had the cow-pock.

"Dr. Jenner gave us the following (four years ago) not one of which have been disproved or contracted.

| | | |
|---|---|---|
| Josiah Mernet | 25 | Years since. |
| Sarah Portlock | 27 | |
| Jn. Phillips | 53 | |
| Mary Barge | 31 | |
| Elizabeth Wynne | 38 | |
| Wm. Stickcomb | 10 | |
| Hester Wakeley | 26 | |

"William Fermor, Esq. of Tusmore, Oxfordshire, the following:

| | | |
|---|---|---|
| Wm. Tredwell | 3 | Years since." |
| Alban Collingridge | 4 | |
| Mr. Stevens | 27 | |
| Thomas Slater | 6 | |
| Mr. H. Collingridge | 10 | |

Many similar facts have been published by Dr. Pearson and others. Dr. Woodville inoculated more than one thousand after cow-pock, not one of whom had small-pox. Such are the arguments the advocates have in support of their opinion; and although there is nothing new in this statement, I have thought some good might arise from repeating them in your Journal, finding, to my regret, that the old adage of, 'None so deaf as those who are unwilling to hear,' is applicable to this subject. I feel it a high moral duty, possessing the sentiments I do of the cow-pock, to offer them again to the senses of those on whom little impression has been made. I would intreat them to lend their ears, their eyes, and their understandings, unbiassed, to a subject in which the community in general, and the low and indigent in particular, are so greatly interested.

 I have

I have very lately (and I reflect on it with the greatest satisfaction) by this mild preventive, rescued a poor mechanic, his wife, and four children, from the terrors of that merciless disease the small-pox; who had left the country (where he found all his exertions inadequate to their support) for this town, where he hopes to be more successful, but where it was next to impossible they should all escape the small-pox; and if one had taken it, it must have communicated to all: — then, how truly deplorable would have been their situation, who would have had to struggle some weeks under the pressure of disease; and, after what is usual (in such cases), parting with their garments and other necessaries, those who survived the wretched wreck of disease would, in all probability, have found themselves in a workhouse or a jail.

And what is the contrast under vaccination? The father has not lost a day's labour, the mother has not been suspended an hour from the numerous duties which such a family of young children must require of her, nor called a minute from other cares to attend to the moans and sufferings of her children!

Can any humane mind be insensible to these advantages? Will any good man *abandon* or *discourage* the progress of a discovery, which, to say *only* what I trust its greatest opposers will confess, has the support and credence of the most enlightened philosophers, experienced practitioners, and philanthropic characters, that adorn and illumine the present gloom and deformity of our hemisphere. I am, &c.

*Copthall Court, July* 14, 1804. L. LEESE.

